# Future Prospects for Clinical Applications of Nanocarbons Focusing on Carbon Nanotubes

**DOI:** 10.1002/advs.202201214

**Published:** 2022-06-26

**Authors:** Naoto Saito, Hisao Haniu, Kaoru Aoki, Naoyuki Nishimura, Takeshi Uemura

**Affiliations:** ^1^ Institute for Biomedical Sciences Interdisciplinary Cluster for Cutting Edge Research Shinshu University 3‐1‐1 Asahi Matsumoto Nagano 390‐8621 Japan; ^2^ Department of Applied Physical Therapy Shinshu University School of Health Sciences 3‐1‐1 Asahi Matsumoto Nagano 390‐8621 Japan; ^3^ Division of Gene Research Research Center for Supports to Advanced Science Shinshu University 3‐1‐1 Asahi Matsumoto Nagano 390‐8621 Japan

**Keywords:** biomaterials, clinical application, nanocarbons, nanomaterials, biosafety

## Abstract

Over the past 15 years, numerous studies have been conducted on the use of nanocarbons as biomaterials towards such applications as drug delivery systems, cancer therapy, and regenerative medicine. However, the clinical use of nanocarbons remains elusive, primarily due to short‐ and long‐term safety concerns. It is essential that the biosafety of each therapeutic modality be demonstrated in logical and well‐conducted experiments. Accordingly, the fundamental techniques for assessing nanocarbon biomaterial safety have become more advanced. Optimal controls are being established, nanocarbon dispersal techniques are being refined, the array of biokinetic evaluation methods has increased, and carcinogenicity examinations under strict conditions have been developed. The medical implementation of nanocarbons as a biomaterial is in sight. With a particular focus on carbon nanotubes, these perspectives aim to summarize the contributions to date on nanocarbon applications and biosafety, introduce the recent achievements in evaluation techniques, and clarify the future prospects and systematic introduction of carbon nanomaterials for clinical use through practical yet sophisticated assessment methods.

## Graphical abstract

Nanocarbons have been applied towards the creation of biomaterials for over 15 years. However, clinical biomaterials employing nanocarbons remain elusive mainly due to concerns of safety. With a particular focus on carbon nanotubes, these perspectives aim to summarize the contributions to date on nanocarbon applications and biosafety evaluation in addition to clarifying the future prospects of clinical nanocarbon applications.

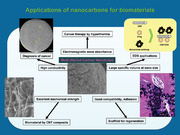

## 1 Introduction

Nanocarbons have been applied towards creating biomaterials for over 15 years, with the number of related papers increasing rapidly in this competitive field.^[^ ^1^, ^2^, ^3^, ^4^, ^5^, ^6^, ^7^, ^8^, ^9^, ^10^, ^11^, ^12^, ^13^, ^14^, ^15^, ^16^, ^17^, ^18^, ^19^, ^20^ ^]^ However, clinical biomaterials employing nanocarbons remain unavailable, largely due to short‐ and long‐term biosafety concerns. Nanocarbon biomaterials may already be applicable in the clinical setting with present technologies provided the successful completion of biosafety assessments.

For over a decade, we have been conducting extensive research on the biomedical applications of nanocarbons, especially carbon nanotubes (CNTs), and have reached the cusp of the world's first clinical use of CNT composite materials. These perspectives will summarize our achievements to date on nanocarbon applications and biosafety, introduce our recent sophisticated evaluation techniques, and clarify the path towards the clinical implementation of nanocarbon biomaterials through current and emerging assessment methods. Although the present report focuses mainly on CNTs,^[^ ^21^, ^22^ ^]^ we have also extensively investigated carbon nanohorns (CNHs),^[^ ^23^, ^24^ ^]^ carbon nanofibers (CNFs),^[^ ^25^, ^26^, ^27^ ^]^ and other nanocarbons.^[^ ^28^ ^]^ Owing to the multitude of properties and experimental conditions for each material, however, additional perspectives are needed to address the full range of nanocarbon materials studied to date.

Biocompatibility and biodegradability are both important considerations in the development of nanobiomaterials. Whereas adequate biocompatibility is essential, biodegradability may be deemed desirable in some cases; many studies exist on such non‐biodegradable nanobiomaterials as nano‐sized hydroxyapatite, nano‐sized gold particles, and nanomagnetic compounds.^[^ ^29^, ^30^, ^31^ ^]^ Nanocarbons exhibit very high biocompatibility in relation to other nanobiomaterials. In contrast, they have low biodegradability.^[^ ^32^ ^]^ These properties may be advantageous in applications where specific effects are desired without the need for biodegradability.

## 2 Progressively Advanced Clinical Applications of Nanocarbon Biomaterials

**Figure 1:**
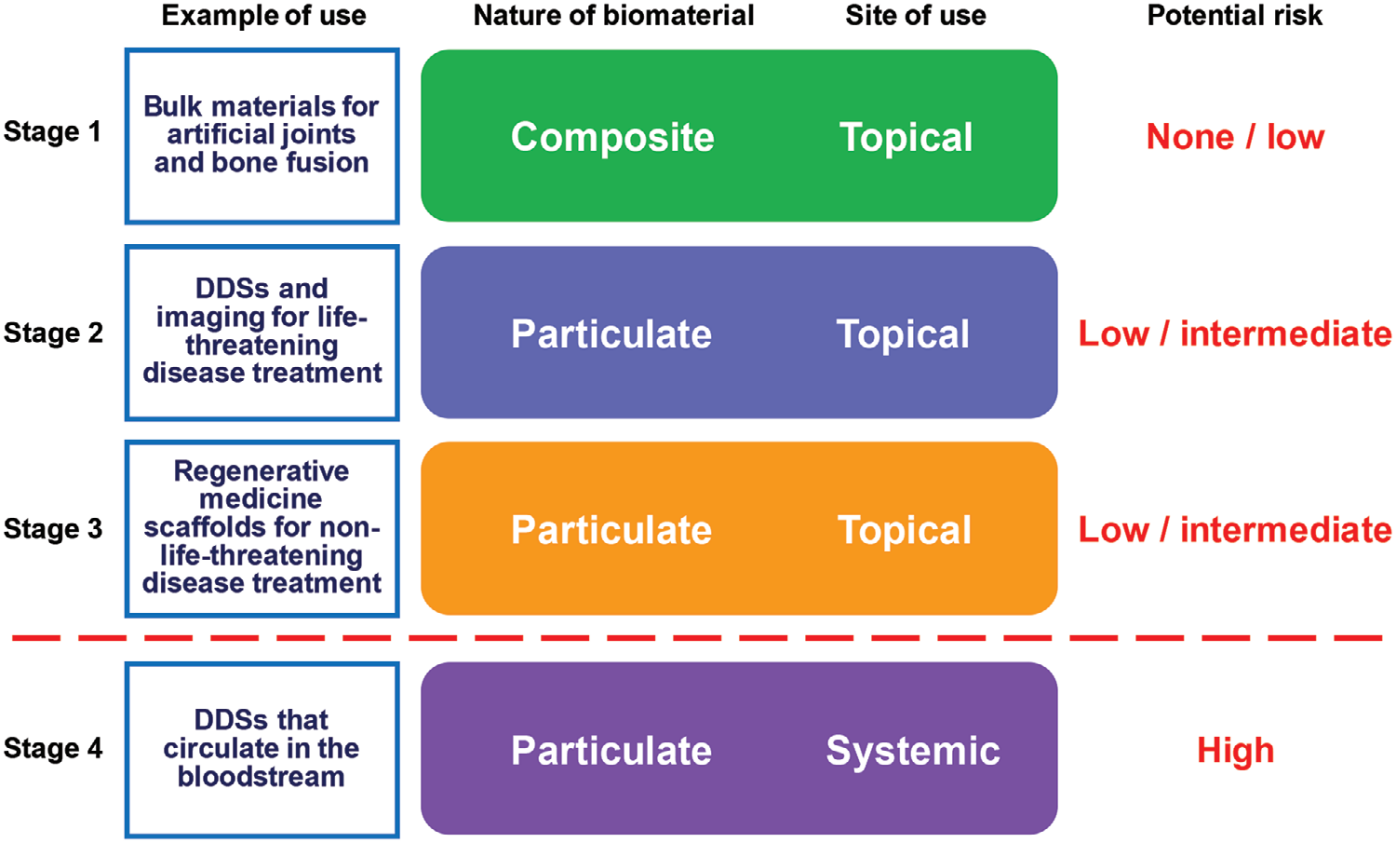
Stages in the clinical application of nanocarbon‐based biomaterials. The medical use of nanocarbons as biomaterials should only progress after demonstrating complete safety at each stage. The decision to proceed to Stage 4 requires extremely careful consideration.

The nanocarbons reported by our group can be broadly considered for four biomaterial applications, which are now being tested for clinical use in a biosafe manner. These are: 1) nanocarbons complexed to a pre‐existing bulk biomaterial and implanted into the body, 2) nanocarbon particles administered locally for life‐threatening diseases, such as cancer, 3) nanocarbon particles given locally for non‐life‐threatening diseases, and 4) nanocarbon particles injected into the circulation for use as a drug delivery system (DDS), imaging modality, or other function (**Figure** **1**).^[^ ^3^ ^]^ At present, applications (1) and (2) are feasible, but their safety in the clinical setting, such as possible accumulation with repeated administration, needs confirmation before proceeding to (3). Although the hurdle of application (4) is high, it will be achievable with sufficient clinical evidence.

### 2.1 Local Implantation of Nanocarbon and Pre‐Existing Bulk Biomaterial Complexes

For this application, nanocarbons are joined with bulk materials used in existing biomaterials to improve therapeutic function. For example, polyethylene used in the sliding part of artificial joints is combined with nanocarbons as a reinforcement material to reduce the amount of wear,^[^ ^33^ ^]^ or collagen used as scaffolds in bone regenerative medicine are complexed with nanocarbons to enhance bone restoration.^[^ ^34^, ^35^, ^36^, ^37^ ^]^ Very few nanocarbons are implanted into the body in such cases. Meanwhile, the pulmonary effects of inhaled nanocarbons, which have been studied worldwide, have been shown as safe depending on the exposure dose.^[^ ^38^, ^39^, ^40^, ^41^, ^42^, ^43^, ^44^, ^45^, ^46^, ^47^, ^48^, ^49^, ^50^ ^]^ Owing to the small overall amount of nanocarbons and the even smaller amount of material released from implanted complexes, nanocarbons are being seriously considered for such applications. Small particle amounts will be tested to assess local tissue safety in addition to the biokinetics of particles when they enter the circulatory system from the local site.

**Figure 2:**
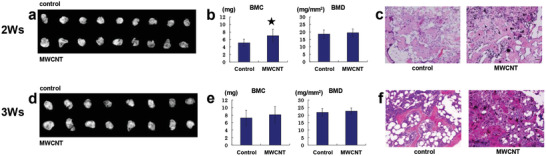
MWCNTs accelerate the rate of ectopic bone formation by rhBMP‐2 and collagen. An rhBMP‐2/collagen composite or rhBMP‐2/collagen/MWCNT composite is implanted in mouse back muscle and isolated 2 and 3 weeks later. a) Radiographs taken 2 weeks later. When using a DDS prepared by complexing MWCNTs with collagen (lower panel), larger bone of higher radiography opacity formed than in samples without complexing MWCNTs (upper panel). b) With a DDS consisting of the collagen/MWCNT composite, the ossicles that formed 2 weeks later had a significantly higher bone mineral content than those with collagen alone. c) Histological profile of ossicles isolated 2 weeks later. A thicker and denser trabecula had formed with the collagen‐MWCNT composite than with collagen alone. d) Three weeks later, there are no differences in radiographic bone opacity or size between the two groups. e) Three weeks later, there are no significant differences in the mineral content or density of the newly formed bone between the two groups. f) Histological profile of the ossicles isolated 3 weeks later. In both cases, the bone tissue is normal, consisting of normal trabecula and hematopoietic bone marrow, with no remarkable differences between them. In the MWCNT composite tissue, the MWCNTs are incorporated uniformly into the trabecula and bone marrow. The MWCNTs had entered the trabecula and bound directly to the bone. *p < 0.05 between samples with and without CNTs (unpaired Student's *t*‐test). Hematoxylin‐eosin staining. Original magnification ×20. Adapted with permission.^[^ ^37^ ^]^ Copyright 2008, Wiley‐VCH.

As an example of biokinetic assessment, our earlier study investigated osteogenesis promoted in vivo using a collagen composite with multi‐walled CNTs (MWCNTs), which have been reported to promote osteoblast proliferation in vitro (**Figure** **2**). By intramuscularly implanting a collagen composite of MWCNTs as a DDS for recombinant human bone morphogenetic protein‐2 (rhBMP‐2) into laboratory animals, the MWCNTs did not inhibit bone formation, but rather bound directly to the bone tissue and became incorporated into the bone. Moreover, the MWCNT‐collagen composites did not induce inflammatory reactions in the muscle, causing little biological responses to the foreign substance. When complexed with MWCNTs, collagen promoted bone formation by BMP to a greater degree than collagen alone, with no such adverse effects as osteolysis owing to good bone tissue affinity. This was the first evidence of MWCNTs promoting bone formation in vivo. Since then, there have been rapid advances in their applications with bone‐related biomaterials and bone regenerative medicine worldwide.^[^ ^37^ ^]^

We have extensively studied the reactions of bone‐forming osteoblasts and bone‐degrading osteoclasts to MWCNTs in order to elucidate the mechanism of bone formation promotion by MWCNTs. First, MWCNTs attract calcium to raise calcium concentrations around osteoblasts. Undifferentiated osteoblasts sense the elevation of calcium concentration and differentiate into mature osteoblasts. The differentiated osteoblasts begin osteogenesis, and in this process, a large amount of alkaline phosphatase (ALP) is released in the surrounding area. As ALP concentrations rise, calcification is induced around the MWCNTs. Repeated MWCNT‐osteoblast interactions are considered to synergistically accelerate calcification and promote osteogenesis. Meanwhile, undifferentiated osteoclasts take up MWCNTs intracellularly to suppress the nuclear transfer of the NFATc1 transcription factor, thereby suppressing osteoclast differentiation. In addition to the biochemical interactions of MWCNTs with osteoblasts and osteoclasts, other factors may be involved in the mechanism of osteogenesis promotion by MWCNTs. In particular, cell adhesion to MWCNTs, vascular invasion around MWCNTs, and the relationships between MWCNTs and collagen molecules should all be further investigated.^[^ ^51^, ^52^ ^]^

### 2.2 Administration of Nanocarbon Particles Locally in Life‐Threatening Diseases

The next application step has been the topical administration of nanocarbons in the form of particles. In this role, nanocarbons are made to exhibit their unique utility in DDSs and other applications. Such clinical uses preferentially commence testing for life‐threatening diseases, such as cancer. First, a careful safety evaluation of particles in the local tissue and biokinetic assessment in the circulatory system of particles escaping via capillaries or lymphatic vessels are carried out. Once the systemic examinations reveal the organ(s) into which particles are deposited, local safety assessments of the affected tissues are performed in the same way as in the implanted tissue, albeit for the expectedly smaller amounts of nanocarbons. Confirming all safety aspects and achieving clinical application will significantly alter the way potentially fatal diseases are treated and greatly advance the world's medical science.

**Figure 3:**
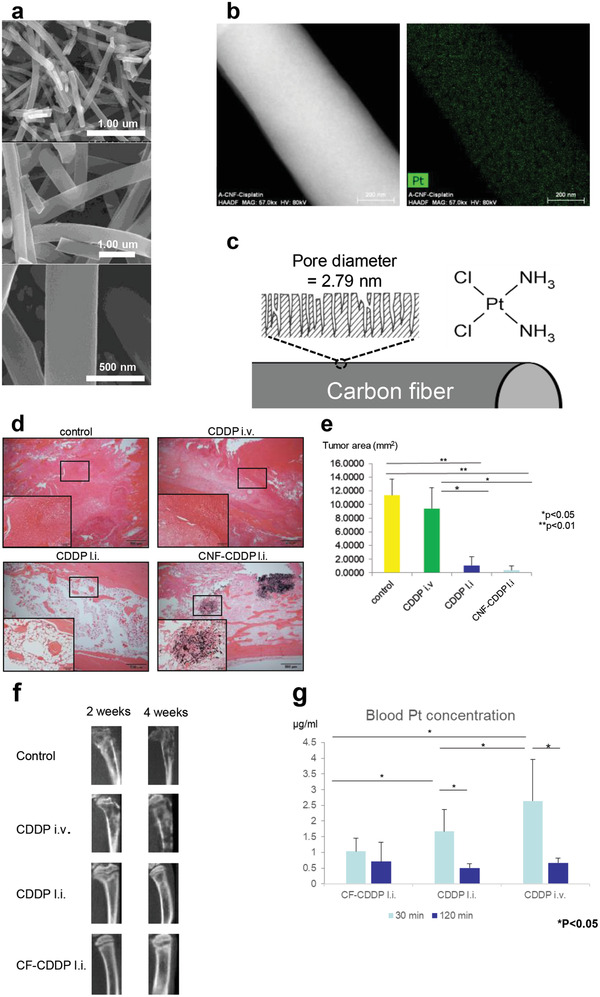
a) The structure of the CF‐CDDP is identified using a scanning electron microscope (SEM) as a fiber‐entangling columnar structure of 400 nm in diameter and 20–100 µm in length. b) Surface distribution of platinum atoms. Transmission electron microscopy‐energy dispersive X‐ray spectroscopy showed that the platinum atoms are distributed uniformly on the CF surface. c) A large number of pores of 2.79 nm in mean diameter are open on the CF‐CDDP surface, with cisplatin present on the surface and inside pores. d) Tissue from a model of cancer metastasis to the bone prepared by injecting Walker 256 breast cancer cells into the rat tibia is examined in the median sagittal plane using a light microscope. Hematoxylin‐eosin staining. In the control group and CDDP i.v. group, tumor tissue showed high occupation in the marrow cavity. In the CDDP l.i. group, tumor tissue is scant, whereas considerable fatty marrow is seen. In the CNF‐CDDP l.i. group, there are almost no tumor cells or fatty marrow, although remarkable fibrous bone tissue is noted around the CNF. e) Measured area of a cancer cell region in rat tibia tissue in the median sagittal plane (*n* = 5). Cancer is not suppressed in the CDDP i.v. group. In both the CDDP l.i. group and the CNF‐CDDP l.i. group, an anticancer effect is evident. f) The anticancer effect is evaluated by µCT using tibial images in the median sagittal plane. An anticancer effect is not noted in the CDDP i.v. group, but is detectable in the CDDP l.i. group and the CNF‐CDDP l.i. group. g) Blood platinum concentrations. Administration of Walker 256 breast cancer cells to the rat tibia is followed by local injections of CNF‐CDDP and CDDP and i.v. injection of CDDP 2 days later (*n* = 5). Blood is collected via the caudal vein 30 and 120 min after drug administration. Platinum atoms in the CDDP composite are measured using atomic absorption spectroscopy. In the CNF‐CDDP l.i. group, post‐dose blood platinum concentration is significantly lower and showed the least change over time. At 120 min post‐dose, no significant differences in blood platinum concentration are found among the groups. Reproduced under the terms of the Creative Commons CC‐BY license.^[^ ^53^ ^]^ Copyright 2020, Royal Society of Chemistry.

To explain such an application, the use of CNFs supplemented with an anticancer agent for cancer‐metastasized bone was examined, as described below.^[^ ^53^ ^]^ When cancer has metastasized to the bone, the patient's quality of life decreases largely due to pain and functional impairment from bone destruction.^[^ ^54^, ^55^, ^56^, ^57^, ^58^, ^59^, ^60^, ^61^, ^62^, ^63^, ^64^, ^65^ ^]^ Traditionally, DDSs for cancer treatment have been prepared with biodegradable materials; however, the cancer‐metastasized bone environment varies widely and lacks order, and the timing of sustained drug release and other factors cannot be controlled satisfactorily with the use of conventional materials. CNFs are not biodegradable and can carry a wide variety of molecular species when processed to have pores on their surfaces. The CNFs used in our recent study were nearly nano‐sized particles of 400 nm in diameter (**Figure** **3**). We developed a novel control system for the cancer‐metastasized bone environment by complexing the CNFs into an anticancer agent. To first introduce cisplatin (CDDP) into CNF surface pores, the CNFs were heated along with KOH to create pores of 2.8 nm in diameter. At a CNF‐specific surface area of 3253 m^2^ g^−1^, pore volume of 2.27 cm^3^ g^−1^, and CNF:CDDP mass ratio of 3:10, we succeeded in efficiently filling the CNF pores with CDDP in this case of hydrophobic CDDP and hydrophobic CNF pores. When using other anticancer agents, some of which are hydrophilic and highly variable in polarity, the cancer‐metastasized bone environment should also be taken into account. The prepared sample was successfully tested on target breast cancer cells in vitro and demonstrated cancer cell suppression effects. In a subsequent experiment, an in vivo bone model of cancer metastasis was prepared by injecting breast cancer cells into the rat tibia. The sample was injected into the same site, and images and tissue specimens were evaluated over time to quantify the cancer tissue suppression effect. Longitudinal blood measurements of the anticancer agent were taken as well. When an injection of breast cancer cells was followed by the administration of CDDP at 10 mg kg^−1^ (intravenously (i.v.) or locally (l.i.)) or CNF‐CDDP at 10 mg kg^−1^ (l.i.), similar cancer tissue proliferation was observed in the CDDP (i.v.) group and the control group, whereas proliferation was highly suppressed in the CDDP (l.i.) group and the CNF‐CDDP (l.i.) group. On the other hand, the blood CDDP concentration in the CDDP (l.i.) group was significantly higher than in the CNF‐CDDP (l.i.) group. This study showed that CNF‐CDDP had similar anticancer properties in the cancer‐metastasized bone environment to CDDP alone and was less likely to release anticancer agents into the blood and cause adverse reactions. Future topical applications of CNF‐CDDP for cancer‐metastasized bone are expected to effectively suppress cancer with lower incidences of adverse events.

### 2.3 Administration of Nanocarbon Particles Locally in Non‐Life‐Threatening Diseases

Nanocarbon particles may be of clinical use for the treatment of non‐life‐threatening diseases, such as diabetes mellitus,^[^ ^66^, ^67^, ^68^, ^69^, ^70^, ^71^, ^72^, ^73^, ^74^, ^75^, ^76^, ^77^, ^78^, ^79^, ^80^, ^81^, ^82^, ^83^, ^84^, ^85^ ^]^ and in bone regenerative medicine.^[^ ^27^, ^33^, ^86^, ^87^, ^88^ ^]^ The safety assessment approaches are exactly the same as in application (2), although the principle of therapeutic effect versus risk requires much more stringent standards. Once the evaluation methods for application (2) have been established, the expanded safety testing of a wide variety of nanocarbon uses in clinical practice will surely proceed to clinical use.

**Figure 4:**
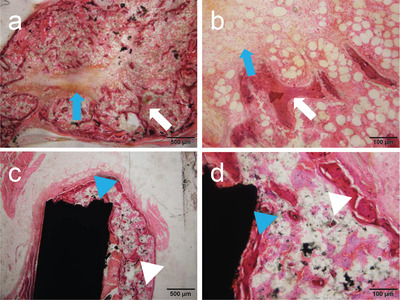
Histological image at 3 weeks after implantation of rhBMP‐2‐containing MWCNT blocks in mouse back muscle. a,b) Ectopic bone formed around an rhBMP‐2‐containing PET‐fiber‐reinforced collagen sheet. Blue arrow: remnant of the PET‐fiber‐reinforced collagen sheet. White arrow: trabecular structure. c,d) Ectopic bone formed around an rhBMP‐2‐containing MWCNT block. The MWCNT block and newly formed bone are bound together firmly. Although the formed bone is found to contain a small amount of MWCNTs, no MWCNTs are detected in the surrounding tissue along with no inflammatory reactions. Blue arrowhead: MWCNT block. White arrowhead: trabecular structure. Hematoxylin‐eosin staining. Reproduced under the terms of the Creative Commons CC‐BY license.^[^ ^86^ ^]^ Copyright 2017, The Authors. Published by PLOS.

An example of such an application is an engineered 3D MWCNT block serving as a scaffold for bone tissue regeneration (**Figure** **4**). In recent years, many studies have used composite materials that contained MWCNTs with high bone affinity as a scaffold in bone regenerative medicine as described in application (1); however, none have reported on constructing scaffolds using MWCNTs alone. In our experiment, MWCNT blocks were prepared in a 3D structure that maximized mechanical strength, and their efficacy as a scaffold for bone defect repair was assessed.^[^ ^86^ ^]^ When the MWCNT blocks were supplemented with rhBMP‐2 and implanted into mouse back muscle, bone formed in close contact with the blocks at a mass similar to that obtained using collagen sheets, the gold standard for clinically used scaffolding in bone regeneration. With its adequate mechanical strength, the MWCNT block may serve as a spacer to fill bone defects and as a scaffold for bone regeneration induced by rhBMP‐2.

### 2.4 Injection of Nanocarbon Particles into the Circulatory System as a DDS or for Imaging in Life‐Threatening Diseases

Since the risk of this application is higher than those of applications (1) to (3), it will be necessary to carry out safety evaluations very carefully through intravenous administration in experimental animals to assess all organs in which nanocarbons accumulate. Unlike that for local escape, the amount of nanocarbons entering the circulatory system is considerably greater in application (4), thus requiring stringent biokinetic assessment. Recent advances in nanoparticle dispersal technology have revealed that organ accumulation is considerably less than previously expected,^[^ ^89^, ^90^, ^91^ ^]^ although target sites still need examination using adequate particle amounts. The development and successful clinical application of such safety assessment methods will lead to a therapeutic revolution for life‐threatening diseases. Further research is required to ascertain whether nanocarbon particles have greater potential than other probes. For example, nanocarbons offer several key advantages, including the simultaneous addition of markers and anticancer agents to their surfaces owing to their high surface reactivity.

## 3 Basic Technologies for Safety Assessment

It will be imperative to establish reliable techniques for evaluating the safety of nanocarbons to proceed with the above clinical applications. The safety assessment of nanocarbons and pre‐existing bulk‐biomaterial composites may be performed according to International Organization for Standardization (ISO) guidelines, but will very likely be considered compliant; the quantity of nanocarbons is small and they barely decompose, and so testing results are expected to reflect those of the parent material. However, the biosafety assessment of nanocarbons alone in adequate doses is warranted if any nanocarbons are suspectedly released, even for nanocarbon and bulk‐material complexes. What we are clarifying at present in both cellular and animal studies are: 1) appropriate controls and 2) the dispersibility of nanocarbons. In animal studies, 3) biokinetic evaluation is important, for which we have developed various examination techniques have been proposed. Most importantly, clarifying 4) carcinogenicity is a key factor regarding the biological applications of nanocarbons in living organisms.

Many published studies of in vivo nanocarbon experiments have used rodent animal models. Moving forward, careful discussion and stringent standards will be required to apply experimental systems on the human body. For this reason, a systematic approach to confirm the safety of nanomaterials in a step‐by‐step process prior to clinical application is needed, such as beginning with a composite and then moving to nanoparticle use at localized sites in life‐threatening diseases.

### 3.1 Appropriate Controls

Neither nanocarbons nor many nano‐sized biomaterials have been clinically applied to date, mainly due to a lack of established safety assessment controls. Most chemical substances have standard positive and negative controls as stipulated by ISO guidelines, and there are clear positive and negative controls for bulk biomaterials. However, there are as yet no control standards for the biosafety evaluation of nano‐sized materials. Hence, although the Organisation for Economic Co‐operation and Development proposes that nanomaterials be evaluated in accordance with chemical substances, there is a general consensus that it is impossible to treat nanomaterials and chemicals equally. Fortunately, there exists an optimal negative control for nanocarbons: the highly purified carbon black that is a constituent of black tattoos. Black tattoos have been introduced into the human body since ancient times and have been demonstrated as safe in countless cases worldwide.^[^ ^92^, ^93^, ^94^, ^95^, ^96^ ^]^ Moreover, there is no ambiguity on whether to make the mass, volume, or number of particles constant when comparing two materials, which is a problem with nanoparticles. Since nanocarbons are allotropic to carbon black, they can be easily compared on a mass‐based basis; indeed, a significant number of papers have already used carbon black as negative controls.^[^ ^51^, ^52^, ^97^, ^98^, ^99^, ^100^, ^101^, ^102^, ^103^, ^104^, ^105^, ^106^ ^]^ Unfortunately, positive controls have not yet been identified for nanocarbons, although the use of ISO‐specified positive controls for chemicals can be acceptable to ensure that the experimental system is functioning.

**Figure 5:**
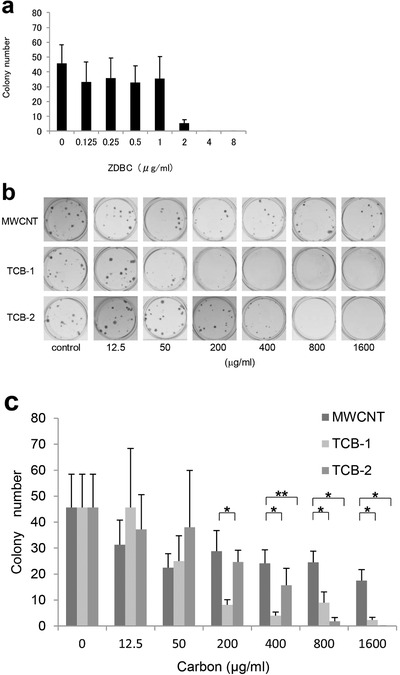
The cytotoxicity of MWCNTs is equivalent to that of the carbon black used in tattoos. a) Validity of cytotoxicity evaluation in a colonization test. The colonization capacity of V79 cells (Chinese hamster lung fibroblast line JCRB0603) decreased in a concentration‐dependent manner in the presence of the positive control ZDBC, and the 50% inhibitory concentration for colony count (reference value range: 1–4 µg mL^−1^) is between 1.56 and 3.12 µg mL^−1^. The cytotoxicity of the test substance is therefore found to be evaluated properly. b) Photomicrographs from the colonization test by the direct contact method. The V79 cell colony count with the culture broth alone is compared with the colony counts with an MWCNT solution as well as two carbon black solutions for tattoos (TCB‐1 solution and TCB‐2 solution, respectively). The concentrations of the solutions are 12.5, 50, 200, 400, 800, and 1600 µg mL^−1^, respectively. c) Colony formation of the MWCNT, TCB‐1, and TCB‐2 solutions by concentration. MWCNTs inhibited colony formation concentration‐dependently, as did both TCB‐1 and TCB‐2. A significantly larger colony number is observed in the MWCNT group than in the TCB‐1 group at 200 mg/mL or more. In comparisons of MWCNTs and TCB‐2, the colony number of MWCNTs is significantly larger at 400 mg mL^−1^ or more. Error bars indicate the standard deviation (*n* = 6). *p < 0.001. **p = 0.016. Adapted with permission.^[^ ^97^ ^]^ Copyright 2011, Elsevier.

To date, we have conducted several short‐ and long‐term implantation studies in vivo as well as an in vitro cytotoxicity study of MWCNTs and evaluated their biological safety in terms of the most fundamental parameters using the two kinds of carbon black in actual use in tattoos as reference materials (**Figure** **5**).^[^ ^97^ ^]^ All studies demonstrated that MWCNTs were highly safe to living organisms at levels equivalent to tattoos.

### 3.2 Dispersibility

A major advancement in recent nanocarbon safety assessment has been techniques for dispersing nanocarbons in solutions. Improvements in the performance of nanoparticle dispersants and dispersion equipment have allowed some nanocarbons to disperse almost completely. A clear difference has been found between the results of previous safety studies using nanocarbons aggregated due to poor dispersibility and the findings with well‐distributed nanocarbons. For example, several initial investigations examining the biokinetics of CNTs in the circulatory system of mice found that the organs in which CNTs accumulated differed widely to include the liver, spleen, lungs, and kidneys. However, the use of well‐distributed CNTs surprisingly revealed the pancreas as the most likely place for accumulation, with little other organ involvement.^[^ ^89^ ^]^ Taken together, greater emphasis should be placed on the results of recently established well‐distributed nanocarbon safety assessments in both cellular and animal studies.

**Figure 6:**
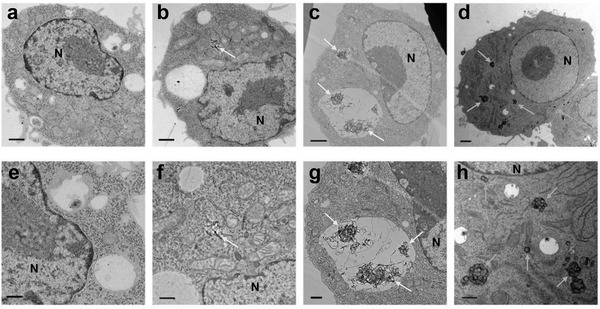
TEM images of RAW264 cells exposed to MWCNTs and CNHs for 24 h. a,e) Control, b,f) high‐dispersion MWCNTs, c,g) low‐dispersion MWCNTs, and d,h) high‐dispersion CNHs. a–d) are shown at low magnification (scale bar: 1 µm). (e–h) are shown at high magnification (scale bar: 500 nm). Yellow arrow: MWCNTs. Green arrow: CNHs. N: nucleus. Only a small proportion of the cells exposed to high‐dispersion MWCNTs incorporated the MWCNTs, which are present in the cytoplasmic matrix without being vesiculated (b,f). Low‐dispersion MWCNTs are entangled intracellularly in lysosomes (c,g). High‐dispersion CNHs are present in a range of forms, from single particles to several dozens of aggregate particles in the cytoplasmic matrix (d,h). Adapted with permission.^[^ ^110^ ^]^ Copyright 2018, Dove Press.

We also investigated the influence of dispersants on MWCNT uptake into cells and revealed that the uptake of nanocarbons differed according to the dispersant used.^[^ ^107^ ^]^ This finding indicated that variability in the dispersants used in safety assessments might change the evaluation results of a given material. A standard ordinary water‐bath sonicator was originally used for dispersing MWCNTs. However, although high‐output sonication could increase MWCNT dispersibility, it also induced molecule cleavage; the MWCNTs were not necessarily dispersed in a monofilament or monoparticle state.^[^ ^108^ ^]^ When the ultimate goal is to deliver nanocarbons into the circulatory system, it is necessary to evaluate them in a fully dispersed state without morphological changes. A high‐output sonicator that avoids local ultrasonic concentration by rotating the sample vial was developed to achieve this aim. We used this device to disperse MWCNTs and CNHs, confirm adequate dispersibility, and then expose the nanomaterials to the cell lines of interest.^[^ ^109^, ^110^ ^]^ Among the well‐dispersed nanocarbons, particulate CNHs were incorporated into macrophage‐like cells, while fibrous MWCNTs were scarcely incorporated into macrophage‐like cells or bronchial epithelium‐derived cells (**Figure** **6**). On the other hand, MWCNTs with insufficient dispersion became incorporated into macrophage‐like cells in large amounts. The rotary high‐output sonicator used in our studies is expected to become a standard instrument in the future as it ensures sufficient dispersion without physically damaging the nanocarbons.

### 3.3 Biokinetic Evaluation

The methods for evaluating biokinetics to date include preparing and observing tissue sections,^[^ ^111^, ^112^ ^]^ attaching contrast materials to surfaces for labeling,^[^ ^113^, ^114^, ^115^ ^]^ carbon isotopes,^[^ ^116^, ^117^, ^118^ ^]^ and Raman's assay.^[^ ^119^, ^120^, ^121^, ^122^, ^123^ ^]^ However, such problems as quantitative limitations in preparing and observing tissue sections, changes in the properties of nanocarbons, and the possibility of contrast material detachment from surfaces exist. Nanocarbons made from carbon isotopes are labor‐intensive and technically demanding, and although Raman analyses can detect some nanocarbons, they are not applicable for many others.

**Figure 7:**
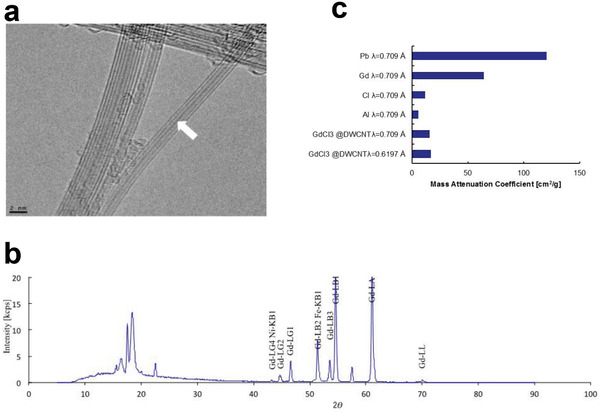
Synthesis and characterization of gadolinium peapods. a) TEM picture of gadolinium peapods. Gadolinium trichloride (GdCl_3_) particles are doped in the hollow part of the inner CNT (white arrow). b) X‐ray fluorescence analysis spectrum of gadolinium peapods. The mass ratio of carbon and gadolinium are 91.3770 and 2.1016 mass%, respectively. c) Mass attenuation coefficient of a gadolinium peapod. The mass attenuation coefficients of lead, gadolinium, chlorine, and aluminum are obtained from the literature. Reproduced under the terms of the Creative Commons CC‐BY license.^[^ ^124^ ^]^

In order to solve these shortcomings, assessment methods considering the characteristics of specific nanocarbons have been developed.^[^ ^124^ ^]^ For example, we have published a technique in which contrast medium molecules are placed in a hollow CNT cavity for dynamic assessment with conventional imaging equipment. The CNTs were called “peapods” due to the ordered arrangement of atoms, molecules, or particles in the central space. It was possible to observe biodistribution by imaging peapods containing contrast materials in biokinetic assessments. The main advantages of this method are that the contrast material does not fall out of the peapod and that biokinetics can be evaluated under the conditions of unaltered CNTs. To evaluate the biodistribution of CNTs, we prepared peapods by labeling with a heavy metal for biokinetic assessment using MRI or CT (**Figure** **7**). Specifically, double‐wall CNTs containing gadolinium trichloride enabled gadolinium detection by MRI. Most recently, we have succeeded in developing CT‐based evaluation of biokinetics using platinum in CNTs.^[^ ^125^ ^]^ The novel peapod method has allowed us to evaluate systemic distribution consistently, conveniently, and without altering the surface properties of the target CNTs, even at a non‐specialized facility.

Recently, measurements using infrared irradiation have greatly improved the detection of single‐walled CNTs. It will also be necessary to combine multiple evaluation methods, such as examining downstream organs after elucidating the systemic distribution of nanocarbons.

**Figure 8:**
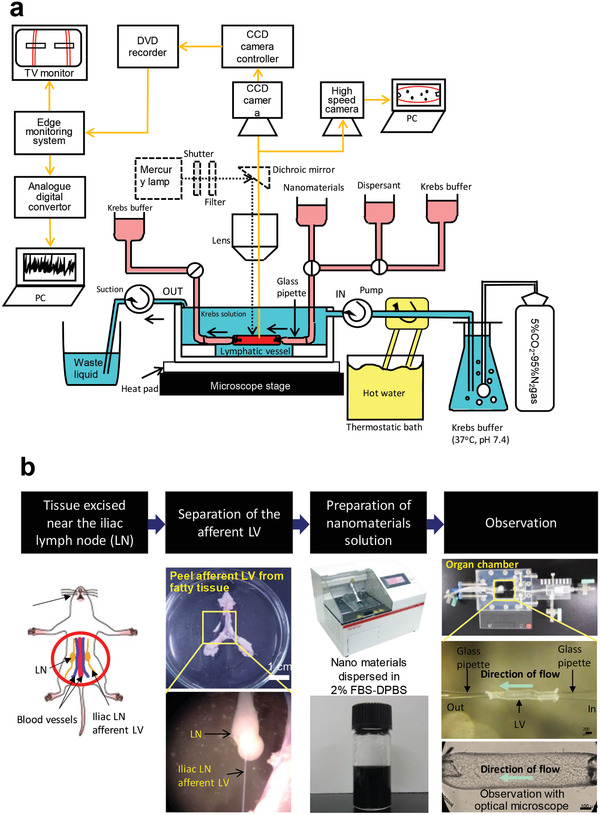
a) Schematic diagram of the entire isolated lymphatic vessel cavity perfusion system. The system comprises an isolated rat lymphatic vessel, an organ chamber to immobilize the vessel, a light microscope to examine the lymphatic vessel and nanomaterial being perfused into the vessel, a CCD camera, a TV monitor, a DVD recorder, a lymphatic vessel tracker, and a computer. The lymphatic vessel is shown in red, perfusion in the lymphatic vessel is in pink, and perfusion outside the lymphatic vessel is in blue. Krebs’ solution perfused in the lymph vessel is aerated with a gaseous mixture of 5% CO_2_ and 95% N_2_ to reproduce the anaerobic conditions in vivo. To maintain the chamber's internal temperature between 37.5 and 38.5 °C, the perfusing solution is warmed using a constant‐temperature vessel with a plate heater placed under the chamber. b) Procedure of setting lymphatic vessels. The method for isolating rat tissue around lymphatic vessels and joining a lymphatic vessel to the chamber is as follows: anesthetized Wistar rats are exsanguinated via the axillary artery and laparotomized, after which the iliac lymph nodes and surrounding lymphatic vessels, arteries, veins, and adipose tissue are ligated together and isolated. An iliac lymph node afferent lymphatic vessel is isolated from adipose tissue in a Petri dish filled with Krebs’ solution and cut into a piece of 2–3 mm length. Both ends of the lymphatic vessel piece are cannulated to a glass micropipette in the chamber and immobilized by suture ligation. A homogeneous dispersion of nanomaterials to be perfused in the lymphatic vessel is prepared by sonication. While applying an inside pressure of 6 cm H_2_O to the specimen cavity and perfusing the outside of the specimen with Krebs's buffer solution (pH 7.4, 37.5–38.5 °C) aerated with 5% CO_2_ and 95% N_2_, the lymphatic vessel is induced to contract spontaneously, and the buffer solution and nanomaterial dispersion are perfused into the cavity. LV: lymphatic vessel, LN: lymphatic node. Adapted with permission.^[^ ^126^ ^]^ Copyright 2021, Elsevier.

Further to biodistribution, we have investigated the biokinetics of CNTs in detail. In one study, we prepared an isolated lymphatic vessel cavity perfusion system for analyzing the motion of nanocarbons in lymphatic vessels and the corresponding vessel responses (**Figure** **8**).^[^ ^126^ ^]^ Having reached biological tissue via the bloodstream after i.v. injection or direct administration to subcutaneous tissue, tumors, and other lesions, nanocarbons are known to enter lymphatic vessels and migrate through the lymphatic system or accumulate in lymph nodes. Therefore, importance should be placed on elucidating the biokinetics of the interactions of lymphatic vessels with nanocarbons. Our novel isolated lymphatic vessel cavity perfusion system enabled us to visually and quantitatively clarify the interplay between nanocarbons and lymphatic vessels towards establishing a new method of evaluating the biosafety of nanocarbons. We could successfully perfuse nanocarbons into isolated rat lymphatic vessels in vitro and comprehensively evaluate nanoparticle motion, spontaneous contractions and other responses of the lymphatic vessels, and histological vessel profiles (Video S1, Supporting Information). With this system, important information on nanocarbon biokinetics can be obtained and contribute significantly to a wide variety of clinical applications. The lymph vessel experiments described here are also planned for application in larger non‐rodent mammals. Furthermore, this experimental system is highly versatile and is even applicable in human samples provided that all ethical issues are addressed.

### 3.4 Carcinogenicity

**Figure 9:**
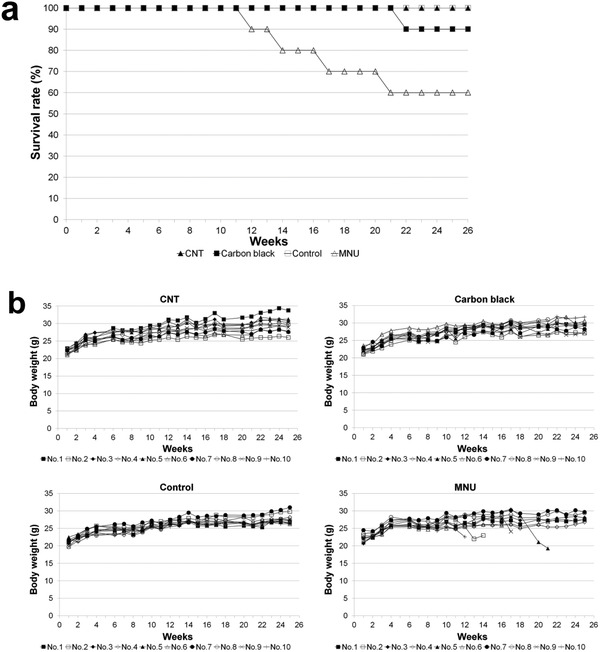
Survival rates and body weight changes in spontaneously cancer‐developing rasH2 mice. a) Mouse survival rates. All mice in the CNT group are alive at week 26. In the carbon black group, 1 animal died at week 22, and 9 of the 10 animals survived at week 26. In the solvent‐only group, all mice are alive at week 26. In the *N*‐methyl‐*N*‐nitrosourea (MNU) group, 1 animal died at weeks 13, 14, 17, and 22, and 6 of the 10 animals are alive at week 26. b) Changes in animal body weight. Body weight changes are similar among the CNT group, carbon black group, and solvent‐only group. Body weight began to decrease with time from week 12 in the MNU group. Adapted with permission.^[^ ^128^ ^]^

The most critical issue regarding the biosafety of nanocarbons is whether or not they induce tumors in various organs. In previous reports, no tumors were observed in accumulating organs when nanocarbons were introduced into the circulatory system of normal laboratory animals. However, inhaled nanocarbons have received mainstream attention for their carcinogenicity, with CNTs also being reported to form tumors when administered intraperitoneally to laboratory animals.^[^ ^114^ ^]^ Thus, the most stringent conditions are needed for carcinogenicity testing. We recently examined tumorigenesis using a genetically modified carcinogenic rasH2 mouse line that has been recognized by the American food and drug administration (FDA) as a carcinogenicity assessment animal for chemical substances.^[^ ^127^ ^]^ MWCNTs were implanted into the subcutaneous tissue of rasH2 mice (**Figure** **9**).^[^ ^128^ ^]^ At 26 weeks post‐implantation, all 10 mice in the MWCNT group and in the solvent‐only group were alive, with only 1 mouse death (10%) in the carbon black group. In the positive control *N*‐methyl‐*N*‐nitrosourea (MNU) group, 6 of the 10 animals were alive at week 26. Similar body weight changes were seen for the MWCNT group, solvent‐only group, and carbon black group. In contrast, the MNU group exhibited marked time‐dependent weight loss from week 12. Histologic examination found a spleen mass in 1 animal in the MWCNT group, which was identified as an inflammatory pseudotumor. No neoplasm was found in the 1 deceased animal in the carbon black group, although an inflammatory pseudotumor was found in the spleen of 1 of the 9 surviving animals. In another animal, a neoplasm had developed in the lung and was histologically diagnosed as adenoma. No neoplasms were found in the solvent‐only group. In the MNU group, neoplasms were detected in all surviving and non‐surviving animals (**Table** **1**). In the MWCNT group, no neoplasm developed at the subcutaneous injection site where macrophage‐phagocytosed CNTs had accumulated, nor were there any inflammatory cells, such as neutrophils and lymphocytes, around the CNTs. Similar findings were witnessed in the carbon black group.

**Table 1:** Incidence of neoplastic changes in resH2 mice following injection with CNTs, carbon black, solvent only, or MNU test substance. Table is modified from a study by Takanashi et al.^[^ ^128^ ^]^

		Control	Carbon black	VGNF	MNU
Organ	Diagnosis/total number	10	10	10	10
Skin (back area)	Papilloma	0	0	0	2
	Keratoacanthoma	0	0	0	0
Skin (other areas)	Papilloma	0	0	0	6
	Keratoacanthoma	0	0	0	0
	Basal cell tumor	0	0	0	0
Spleen	Inflammatory pseudotumor	0	1	0	0
	Hemangioma	0	0	1	0
Hematopoietic system	Malignant lymphoma	0	0	0	2
	Epithelial thymoma	0	0	0	0
Kidneys	Hemangioma	0	0	0	0
Pancreas	Hemangioma	0	0	0	0
Lungs	Adenocarcinoma	0	0	0	0
	Adenoma	0	1	0	1
	Hemangioma	0	0	0	0
Forestomach	Papilloma	0	0	0	10
	Basal cell tumor	0	0	0	0
	Squamous cell carcinoma	0	0	0	0
Oral mucosa	Papilloma	0	0	0	3

## 4 Nanocarbon Biomaterials Expected for Clinical Application in the Near Future

We have received encouragement from the Japanese Pharmaceuticals and Medical Devices Agency^[^ ^129^, ^130^, ^131^ ^]^ (corresponding to the American FDA or the U.K. Medicines and Healthcare Products Regulatory Agency) regarding the progression of CNT composites towards clinical testing. A sample case is described below.^[^ ^33^ ^]^

**Figure 10:**
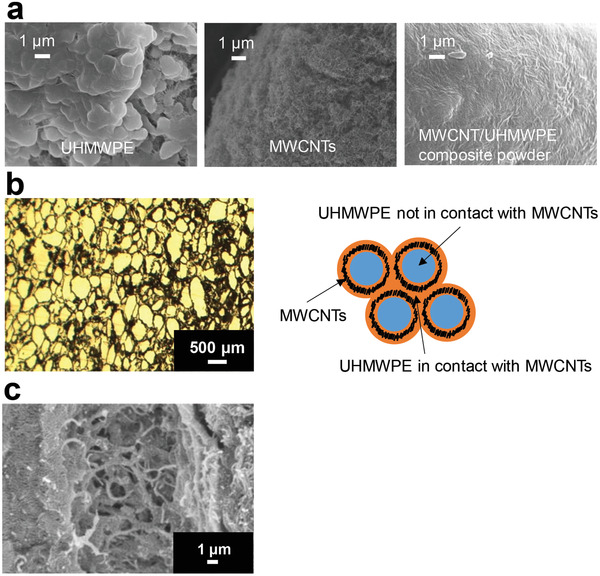
Preparation MWCNT/UHMWPE composites. a) SEM images of UHMWPE, MWCNT, and MWCNT/UHMWPE composite powder kneaded using a twin‐screw extruder with heating. b) When an MWCNT/UHMWPE composite prepared by thermal compression molding of a composite powder of UHMWPE and MWCNT is examined using light microscopy, a honeycomb structure is found comprising laminar MWCNT surrounding a lump of UHMWPE. Left: photomicrograph. Right: diagram of the planar structure. c) SEM image showing that the UHMWPE in contact with the MWCNT had dissolved and bound to the MWCNT. Adapted with permission.^[^ ^33^ ^]^ Copyright 2020, American Chemical Society.

**Figure 11:**
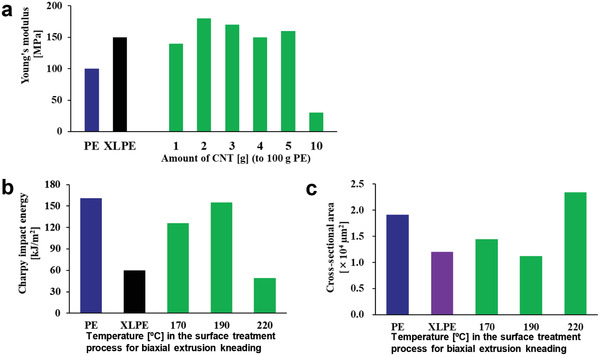
Mechanical properties of MWCNT/UHMWPE composites. PE: UHMWPE. XLPE: crosslinked UHMWPE. a) Tensile testing of MWCNT/UHMWPE composite test pieces showed that Young's modulus is maximized at a 100:2 composition of UHMWPE and CNTs to make the composite harder than crosslinked UHMWPE. b) The temperature in the last stage of processing using a twin‐screw extruder‐kneader (surface treatment step) and the absorption energy of the prepared test piece in the Charpy impact test are measured. The impact absorption energy is maximized at 190 °C processing to a level equivalent to that obtained with non‐MWCNT‐composite UHMWPE. c) In the wear resistance test, cross‐sectional areas with surface wear are measured and evaluated. When the test piece is processed at 190 °C in the surface treatment step of twin‐screw extrusion‐kneading, the wear resistance is maximized to a level equivalent to that of crosslinked UHMWPE. Adapted with permission.^[^ ^33^ ^]^ Copyright 2020, ACS Publications.

**Figure 12:**
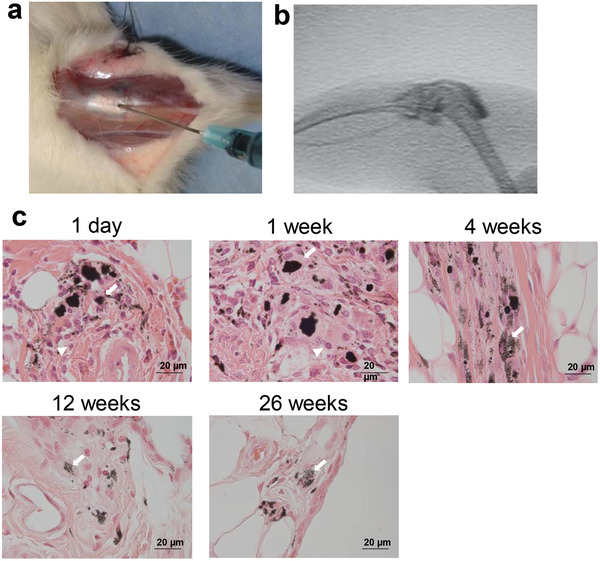
Intra‐articular reactions of MWCNTs alone. MWCNTs of 0.03 mg are dispersed in 80 µL of solution containing a dispersing agent and contrast medium. The dispersion is injected into rat right knee joints for evaluating histological profiles at 1 day, 1 week, 4 weeks, 12 weeks, and 26 weeks after injection (*n* = 4). a) Anterior skin of the rat knee joint is incised, and the MWCNT dispersion is injected into the articular capsule. b) Fluorography at the time of injection confirmed that the MWCNT dispersion did not leak out from the joint. c) Histologically, mildly inflammatory cells are found in the synovial membrane around the MWCNTs on day 1 and at week 1; however, the inflammatory reaction became quiescent at week 4. The MWCNTs are phagocytosed by macrophage‐like cells in the synovial membrane, with some macrophage‐like cells forming an assembly. The histological profiles at weeks 12 and 26 are similar to those at week 4, indicating that no inflammation had occurred over a long period. Scale bar: 20 µm. Adapted with permission.^[^ ^33^ ^]^ Copyright 2020, American Chemical Society Publications.

Since ultrahigh‐molecular‐weight polyethylene (UHMWPE) used in artificial joint sliding elements wears quickly,^[^ ^132^, ^133^, ^134^, ^135^, ^136^, ^137^, ^138^, ^139^, ^140^, ^141^ ^]^ crosslinked UHMWPE is often used in total hip arthroplasty (THA).^[^ ^142^, ^143^, ^144^, ^145^, ^146^, ^147^, ^148^, ^149^, ^150^, ^151^, ^152^ ^]^ However, crosslinked UHMWPE has low impact resistance and is hence easily breakable, with several published cases of breakage in THA.^[^ ^153^, ^154^, ^155^, ^156^, ^157^, ^158^, ^159^ ^]^ For the same reason, crosslinked UHMWPE is avoided for total knee arthroplasty (TKA), which involves the convex contact of joint components. Indeed, artificial joint sliding elements pose the major problem of a trade‐off between wear resistance and impact resistance, and no UHMWPE material has resolved the issue to date. In our study, we addressed this conundrum by complexing UHMWPE with MWCNTs (**Figure** **10**) and evaluating their safety for use in artificial joints. Interestingly, the MWCNT/UHMWPE composites showed wear resistance equivalent to that of crosslinked UHMWPE as well as impact resistance comparable to that of non‐crosslinked UHMWPE (**Figure** **11**). The MWCNT/UHMWPE composites also met all of the criteria for an implantable medical device in a biosafety study based on the ISO10993 series. Taking into account the possible presence of MWCNTs in wear debris, MWCNTs contained in the wear debris of a converted‐for‐rat amount of approximately 1.5 times that expected for 50 years in the worst case were injected into rat knee joints and monitored for 26 weeks. A very mild inflammatory reaction occurred in the joint, but quickly became quiescent (**Figure** **12**). The MWCNTs did not migrate to any other organ. Thus, such MWCNT/UHMWPE composites represent a new biomaterial expected to find safe clinical applications both in THA and TKA as the first artificial joint sliding elements to possess both high wear resistance and high impact resilience. Increased clinical testing of other nanocarbon biomaterials is expected in the near future.

## 5 Outlook

The immense clinical potential of nanocarbons is evident. Optimal controls are being identified, nanocarbon dispersal techniques have evolved, the selection of biokinetic evaluation methods has increased, and carcinogenicity testing under severe conditions is being developed. As the fundamental techniques for the biosafety assessment of nanocarbon biomaterials become more sophisticated, new preparations for clinical applications await. Ultimately, the biosafety of each developed product must be shown in logical and well‐conducted experiments.

Pre‐existing bulk biomaterials composed of nanocarbons will first be clinically applied. Nanocarbon particles will then be employed for the local treatment of cancer and other lethal diseases. Although this may require some encouragement of patient candidates, the expected benefits will be incredible. Encouragingly, a detailed review of previous reports has shown a high probability of success up to this stage. Moreover, the social perception of nanocarbons is changing favorably, and irrefutable evidence is accumulating on their tolerance and safety. It remains unclear at present whether nanocarbons can be administered into the circulatory system, but persistence and impending advancements will surely overcome this hurdle. We believe that the cumulative body of nanocarbon biomaterial research and development, to which numerous researchers have been devoted for over 15 years, will lead to paradigm shifts and major advances in global medicine by following the road towards clinical applications.

## Conflict of Interest

The authors declare no conflict of interest.

## Supporting information

Supporting Information

Supplemental Video 1

Supplemental Video 2

## References

[1] N. Saito , Y. Usui , K. Aoki , N. Narita , M. Shimizu , K. Hara , N. Ogiwara , K. Nakamura , N. Ishigaki , H. Kato , S. Taruta , M. Endo , Chem. Soc. Rev. 2009, 38, 1897. 19551170 10.1039/b804822n

[2] N. Saito , K. Aoki , Y. Usui , M. Shimizu , K. Hara , N. Narita , N. Ogihara , K. Nakamura , N. Ishigaki , H. Kato , H. Haniu , S. Taruta , Y. A. Kim , M. Endo , Chem. Soc. Rev. 2011, 40, 3824. 21487627 10.1039/c0cs00120a

[3] N. Saito , H. Haniu , Y. Usui , K. Aoki , K. Hara , S. Takanashi , M. Shimizu , N. Narita , M. Okamoto , S. Kobayashi , H. Nomura , H. Kato , N. Nishimura , S. Taruta , M. Endo , Chem. Rev. 2014, 114, 6040. 24720563 10.1021/cr400341h PMC4059771

[4] V. Negri , J. Pacheco‐Torres , D. Calle , P. López‐Larrubia , Top. Curr. Chem. 2020, 378, 15. 10.1007/s41061-019-0278-8 31938922

[5] K. Aoki , N. Saito , Nanomaterials 2020, 10, 264. 10.3390/nano10020264 PMC7075247 32033249

[6] K. S. Munir , C. Wen , Y. Li , Adv. Biosyst. 2019, 3, 1800212. 10.1002/adbi.201800212 32627403

[7] M. Samadishadlou , M. Farshbaf , N. Annabi , T. Kavetskyy , R. Khalilov , S. Saghfi , A. Akbarzadeh , S. Mousavi , Artif. Cells, Nanomed., Biotechnol. 2018, 46, 1314. 29043857 10.1080/21691401.2017.1389746

[8] A. Z. Wilczewska , K. Niemirowicz , K. H. Markiewicz , H. Car , Pharmacol. Rep. 2012, 64, 1020. 23238461 10.1016/s1734-1140(12)70901-5

[9] D. A. Gomez‐Gualdrón , J. C. Burgos , J. Yu , P. B. Balbuena , Prog. Mol. Biol. Transl. Sci. 2011, 104, 175. 22093220 10.1016/B978-0-12-416020-0.00005-X

[10] R. Alshehri , A. M. Ilyas , A. Hasan , A. Arnaout , F. Ahmed , A. Memic , J. Med. Chem. 2016, 59, 8149. 27142556 10.1021/acs.jmedchem.5b01770

[11] R. Soleyman , S. Hirbod , M. Adeli , Biomater. Sci. 2015, 3, 695. 26222588 10.1039/c4bm00421c

[12] O. Erol , I. Uyan , M. Hatip , C. Yilmaz , A. B. Tekinay , M. O. Guler , Nanomedicine 2018, 14, 2433. 28552644 10.1016/j.nano.2017.03.021

[13] V. R. Raphey , T. K. Henna , K. P. Nivitha , P. Mufeedha , C. Sabu , K. Pramod , Mater. Sci. Eng., C 2019, 100, 616. 10.1016/j.msec.2019.03.043 30948098

[14] M. A. Saleemi , M. Hosseini Fouladi , P. V. C. Yong , K. Chinna , N. K. Palanisamy , E. H. Wong , Chem. Res. Toxicol. 2021, 34, 24. 33319996 10.1021/acs.chemrestox.0c00172

[15] E. Anaya‐Plaza , A. Shaukat , I. Lehtonen , M. A. Kostiainen , Adv. Healthcare Mater. 2021, 10, 2001162. 10.1002/adhm.202001162 33124183

[16] P. W. Hashim , J. K. Nia , G. Han , D. Ratner , J. Am. Acad. Dermatol. 2020, 83, 1144. 30991121 10.1016/j.jaad.2019.04.020

[17] D. R. Amin , E. Sink , S. P. Narayan , M. Abdel‐Hafiz , L. Mestroni , B. Peña , Molecules 2020, 25, 5189. 10.3390/molecules25215189 PMC7664640 33171802

[18] R. Partha , J. L. Conyers , Int. J. Nanomed. 2009, 4, 261. PMC2789438 20011243

[19] M. Mohajeri , B. Behnam , A. Sahebkar , J. Cell. Physiol. 2018, 234, 298. 30078182 10.1002/jcp.26899

[20] M. S. Kang , H. J. Jang , S. H. Lee , J. E. Lee , H. J. Jo , S. J. Jeong , B. Kim , D. W. Han , Materials 2021, 14, 5104.34501203

[21] S. Iijima , Nature 1991, 354, 56.

[22] S. Iijima , T. Ichihashi , Nature 1993, 363, 603.

[23] S. Iijima , M. Yudasaka , R. Yamada , S. Bandow , K. Suenaga , F. Kokai , K. Takahashi , Chem. Phys. Lett. 1999, 309, 165.

[24] S. Iijima , Phys. B 2002, 323, 1.

[25] C. Kim , K. S. Yang , M. Kojima , K. Yoshida , Y. J. Kim , Y. A. Kim , M. Endo , Adv. Funct. Mater. 2006, 16, 2393.

[26] K. Aoki , Y. Usui , N. Narita , N. Ogiwara , N. Iashigaki , K. Nakamura , H. Kato , K. Sano , K. Kametani , C. Kim , S. Taruta , Y. A. Kim , M. Endo , N. Saito , Small 2009, 5, 1540. 19334009 10.1002/smll.200801610

[27] K. Aoki , H. Haniu , Y. A. Kim , N. Saito , Nanomaterials 2020, 10, 14. 10.3390/nano10030562 PMC7153397 32244931

[28] L. P. Biro , P. Nemes‐Incze , P. Lambin , Nanoscale 2012, 4, 1824. 22080243 10.1039/c1nr11067e

[29] Y. Huang , G. Zhou , L. Zheng , H. Liu , X. Niu , Y. Fan , Nanoscale 2012, 4, 2484. 22371072 10.1039/c2nr12072k

[30] P. J. Kempen , M. F. Kircher , A. de la Zerda , C. L. Zavaleta , J. V. Jokerst , I. K. Mellinghoff , S. S. Gambhir , R. Sinclair , Micron 2015, 68, 70. 25464144 10.1016/j.micron.2014.09.004 PMC4262686

[31] A. Matsumine , K. Takegami , K. Asanuma , T. Matsubara , T. Nakamura , A. Uchida , A. Sudo , Int. J. Clin. Oncol. 2011, 16, 101. 21373775 10.1007/s10147-011-0217-3

[32] Y. Sato , A. Yokoyama , Y. Nodasaka , T. Kohgo , K. Motomiya , H. Matsumoto , E. Nakazawa , T. Numata , M. Zhang , M. Yudasaka , H. Hara , R. Araki , O. Tsukamoto , H. Saito , T. Kamino , F. Watari , K. Tohji , Sci. Rep. 2013, 3, 2516. 23981952 10.1038/srep02516 PMC3755288

[33] A. Sobajima , T. Okihara , S. Moriyama , N. Nishimura , T. Osawa , K. Miyamae , H. Haniu , K. Aoki , M. Tanaka , Y. Usui , K. Sako , H. Kato , N. Saito , ACS Biomater. Sci. Eng. 2020, 6, 7032. 33320600 10.1021/acsbiomaterials.0c00916

[34] E. Hirata , M. Uo , Y. Nodasaka , H. Takita , N. Ushijima , T. Akasaka , F. Watari , A. Yokoyama , J. Biomed. Mater. Res., Part B 2010, 93B, 544. 10.1002/jbm.b.31613 20186828

[35] E. Hirata , M. Uo , H. Takita , T. Akasaka , F. Watari , A. Yokoyama , Carbon 2011, 49, 3284.

[36] E. E. da Silva , H. H. M. Della Colleta , A. S. Ferlauto , R. L. Moreira , R. R. Resende , S. Oliveira , G. T. Kitten , R. G. Lacerda , L. O. Ladeira , Nano Res. 2009, 2, 462.

[37] Y. Usui , K. Aoki , N. Narita , N. Murakami , I. Nakamura , K. Nakamura , N. Ishigaki , H. Yamazaki , H. Horiuchi , H. Kato , S. Taruta , Y. A. Kim , M. Endo , N. Saito , Small 2008, 4, 240. 18205152 10.1002/smll.200700670

[38] R. R. Mercer , A. F. Hubbs , J. F. Scabilloni , L. Wang , L. A. Battelli , S. Friend , V. Castranova , D. W. Porter , Part. Fibre Toxicol. 2011, 8, 21. 21781304 10.1186/1743-8977-8-21 PMC3152886

[39] J. Dong , D. W. Porter , L. A. Batteli , M. G. Wolfarth , D. L. Richardson , Q. Ma , Arch. Toxicol. 2015, 89, 621. 25510677 10.1007/s00204-014-1428-y

[40] F. Samiei , F. H. Shirazi , P. Naserzadeh , F. Dousti , E. Seydi , J. Pourahmad , Environ. Sci. Pollut. Res. Int. 2020, 27, 12096. 31984464 10.1007/s11356-020-07740-5

[41] J. Devoy , H. Nunge , E. Bonfanti , C. Seidel , L. Gaté , F. Cosnier , Nanotoxicology 2020, 14, 1227. 32909484 10.1080/17435390.2020.1814439

[42] G. Oberdörster , V. Castranova , B. Asgharian , P. Sayre , J. Toxicol. Environ. Health, Part B 2015, 18, 121. 10.1080/10937404.2015.1051611 PMC4706753 26361791

[43] J. K. Kim , M. S. Jo , Y. Kim , T. G. Kim , J. H. Shin , B. W. Kim , H. P. Kim , H. K. Lee , H. S. Kim , K. Ahn , S. M. Oh , W. S. Cho , I. J. Yu , Nanotoxicology 2020, 14, 250. 31855090 10.1080/17435390.2019.1700568

[44] E. M. Rydman , M. Ilves , A. J. Koivisto , P. A. Kinaret , V. Fortino , T. S. Savinko , M. T. Lehto , V. Pulkkinen , M. Vippola , K. J. Hämeri , S. Matikainen , H. Wolff , K. M. Savolainen , D. Greco , H. Alenius , Part. Fibre Toxicol. 2014, 11, 48. 25318534 10.1186/s12989-014-0048-2 PMC4215016

[45] P. Kinaret , M. Ilves , V. Fortino , E. Rydman , P. Karisola , A. Lähde , J. Koivisto , J. Jokiniemi , H. Wolff , K. Savolainen , D. Greco , H. Alenius , ACS Nano 2017, 11, 291. 28045493 10.1021/acsnano.6b05652

[46] Q. Wang , Z. Zhao , D. B. Alexander , D. Zhao , J. Xu , H. Tsuda , J. Toxicol. Pathol. 2020, 33, 145. 32764839 10.1293/tox.2019-0075 PMC7396733

[47] [43] S. I. Sinis , C. Hatzoglou , K. I. Gourgoulianis , S. G. Zarogiannis , Front. Physiol. 2018, 9, 295. 29651248 10.3389/fphys.2018.00295 PMC5884948

[48] R. R. Mercer , A. F. Hubbs , J. F. Scabilloni , L. Wang , L. A. Battelli , D. Schwegler‐Berry , V. Castranova , D. W. Porter , Part. Fibre Toxicol. 2010, 7, 28. 20920331 10.1186/1743-8977-7-28 PMC2958975

[49] K. Fujita , M. Fukuda , S. Endoh , J. Maru , H. Kato , A. Nakamura , N. Shinohara , K. Uchino , K. Honda , Toxicol. Lett. 2016, 257, 23. 27259835 10.1016/j.toxlet.2016.05.025

[50] T. Kasai , Y. Umeda , M. Ohnishi , T. Mine , H. Kondo , T. Takeuchi , M. Matsumoto , S. Fukushima , Part. Fibre Toxicol. 2016, 13, 53. 27737701 10.1186/s12989-016-0164-2 PMC5064785

[51] M. Shimizu , Y. Kobayashi , T. Mizoguchi , H. Nakamura , I. Kawahara , N. Narita , Y. Usui , K. Aoki , K. Hara , H. Haniu , N. Ogihara , N. Ishigaki , K. Nakamura , H. Kato , M. Kawakubo , Y. Dohi , S. Taruta , Y. A. Kim , M. Endo , H. Ozawa , N. Udagawa , N. Takahashi , N. Saito , Adv. Mater. 2012, 24, 2176. 22447724 10.1002/adma.201103832

[52] N. Narita , Y. Kobayashi , H. Nakamura , K. Maeda , A. Ishihara , T. Mizoguchi , Y. Usui , K. Aoki , M. Simizu , H. Kato , H. Ozawa , N. Udagawa , M. Endo , N. Takahashi , N. Saito , Nano Lett. 2009, 9, 1406. 19284728 10.1021/nl8030746

[53] T. Kamanaka , H. Haniu , M. Tanaka , T. Takizawa , K. Aoki , M. Okamoto , A. Sobajima , K. Yoshida , H. Ideta , T. Mimura , H. Ishida , K. Ueda , T. Uemura , J. H. Kim , Y. A. Kim , H. Kato , N. Saito , RSC Adv. 2020, 10, 33071. 35515018 10.1039/d0ra05992g PMC9056704

[54] A. Tanaka , H. Katagiri , H. Murata , J. Wasa , M. Miyagi , Y. Honda , M. Takahashi , Bone Joint J. 2020, 102B, 285. 10.1302/0301-620X.102B3.BJJ-2019-0976.R1 32114815

[55] J. J. Meeuse , Y. M. van der Linden , G. van Tienhoven , R. O. B. Gans , J. W. H. Leer , A. K. L. Reyners , G. Dutch Bone Metastasis Study , Cancer 2010, 116, 2716. 20225326 10.1002/cncr.25062

[56] S. Lutz , L. Berk , E. Chang , E. Chow , C. Hahn , P. Hoskin , D. Howell , A. Konski , L. Kachnic , S. Lo , A. Sahgal , L. Silverman , C. von Gunten , E. Mendel , A. Vassil , D. W. Bruner , W. Hartsell , Int. J. Radiat. Oncol., Biol., Phys. 2011, 79, 965. 21277118 10.1016/j.ijrobp.2010.11.026

[57] H. Hara , Y. Sakai , T. Kawamoto , N. Fukase , Y. Kawakami , T. Takemori , S. Fujiwara , K. Kitayama , S. Yahiro , T. Miyamoto , K. Kakutani , T. Niikura , D. Miyawaki , T. Okada , A. Sakashita , Y. Imamura , R. Sasaki , Y. Kizawa , H. Minami , T. Matsumoto , T. Matsushita , R. Kuroda , T. Akisue , J. Bone Oncol. 2021, 27, 100352. 33850700 10.1016/j.jbo.2021.100352 PMC8039818

[58] M. N. Kirkinis , C. J. Lyne , M. D. Wilson , P. F. M. Choong , EJSO 2016, 42, 1787. 27499111 10.1016/j.ejso.2016.03.036

[59] K. Sarahrudi , M. Greitbauer , P. Platzer , J. T. Hausmann , T. Heinz , V. Vécsei , J. Trauma 2009, 66, 1158. 19359930 10.1097/TA.0b013e3181622bca

[60] C. Errani , A. F. Mavrogenis , L. Cevolani , S. Spinelli , A. Piccioli , G. Maccauro , N. Baldini , D. Donati , Eur. J. Orthop. Surg. Traumatol. 2017, 27, 205. 27650452 10.1007/s00590-016-1857-9

[61] R. Wedin , H. C. Bauer , J. Bone Jt. Surg., Br. Vol. 2005, 87, 1653. 10.1302/0301-620X.87B12.16629 16326880

[62] M. Steensma , P. J. Boland , C. D. Morris , E. Athanasian , J. H. Healey , Clin. Orthop. Relat. Res. 2012, 470, 920. 21879407 10.1007/s11999-011-2047-z PMC3270160

[63] Y. Yazawa , F. J. Frassica , E. Y. Chao , D. J. Pritchard , F. H. Sim , T. C. Shives , Clin. Orthop. Relat. Res. 1990, 251, 213. 2295178

[64] N. Harvey , E. R. Ahlmann , D. C. Allison , L. Wang , L. R. Menendez , Clin. Orthop. Relat. Res. 2012, 470, 684. 21879409 10.1007/s11999-011-2038-0 PMC3270182

[65] S. J. Janssen , T. Teunis , F. J. Hornicek , C. N. van Dijk , J. A. Bramer , J. H. Schwab , J. Surg. Oncol. 2016, 114, 507. 27374478 10.1002/jso.24345

[66] I. Ilie , R. Ilie , T. Mocan , F. Tabaran , C. Iancu , L. Mocan , Int. J. Nanomed. 2013, 8, 3345. 10.2147/IJN.S48223 PMC3770514 24039418

[67] D. W. Jiang , Z. S. Liu , K. K. Wu , L. L. Mou , R. Ovalle‐Robles , K. Inoue , Y. Zhang , N. Y. Yuan , J. N. Ding , J. H. Qiu , Y. Huang , Z. F. Liu , Polymers 2018, 10, 375. 10.3390/polym10040375 PMC6415456 30966410

[68] C. M. Ng , H. S. Loh , K. Muthoosamy , N. Sridewi , S. Manickam , Int. J. Nanomed. 2016, 11, 1607. 10.2147/IJN.S98726 PMC4841430 27143882

[69] T. N. H. Nguyen , X. Jin , J. K. Nolan , J. Xu , K. V. H. Le , S. Lam , Y. Wang , M. A. Alam , H. Lee , ACS Biomater. Sci. Eng. 2020, 6, 5315. 33455280 10.1021/acsbiomaterials.0c00647 PMC8203305

[70] X. Nie , Z. J. Chen , L. Pang , L. Wang , H. J. Jiang , Y. Chen , Z. Zhang , C. M. Fu , B. Ren , J. M. Zhang , Int. J. Nanomed. 2020, 15, 10215. 10.2147/IJN.S285134 PMC7751584 33364755

[71] S. Zaman , S. Hussain , F. K. Butt , J. G. Xin , C. J. Zhu , J. Nanosci. Nanotechnol. 2017, 17, 8557.

[72] L. R. Lemmerman , D. Das , N. Higuita‐Castro , R. G. Mirmira , D. Gallego‐Perez , Trends Endocrinol. Metab. 2020, 31, 448. 32396845 10.1016/j.tem.2020.02.001 PMC7987328

[73] R. M. Snider , M. Ciobanu , A. E. Rue , D. E. Cliffel , Anal. Chim. Acta 2008, 609, 44. 18243872 10.1016/j.aca.2007.12.032 PMC2358927

[74] P. W. Barone , M. S. Strano , J. Diabetes Sci. Technol. 2009, 3, 242. 20144355 10.1177/193229680900300204 PMC2771526

[75] W. Liu , X. Zhou , L. Xu , S. Zhu , S. Yang , X. Chen , B. Dong , X. Bai , G. Lu , H. Song , Nanoscale 2019, 11, 11496. 31112195 10.1039/c9nr00942f

[76] M. A. Sá , V. B. Andrade , R. M. Mendes , M. V. Caliari , L. O. Ladeira , E. E. Silva , G. A. Silva , J. D. Corrêa‐Júnior , A. J. Ferreira , Oral Dis. 2013, 19, 484. 23107153 10.1111/odi.12030

[77] Y. Mo , S. Brahmachari , J. Lei , S. Gilead , Y. Tang , E. Gazit , G. Wei , ACS Chem. Neurosci. 2018, 9, 2741. 29986579 10.1021/acschemneuro.8b00166

[78] O. Veiseh , B. C. Tang , K. A. Whitehead , D. G. Anderson , R. Langer , Nat. Rev. Drug Discovery 2015, 14, 45. 25430866 10.1038/nrd4477 PMC4751590

[79] N. M. Iverson , P. W. Barone , M. Shandell , L. J. Trudel , S. Sen , F. Sen , V. Ivanov , E. Atolia , E. Farias , T. P. McNicholas , N. Reuel , N. M. Parry , G. N. Wogan , M. S. Strano , Nat. Nanotechnol. 2013, 8, 873. 24185942 10.1038/nnano.2013.222 PMC4066962

[80] K. Yum , T. P. McNicholas , B. Mu , M. S. Strano , J. Diabetes Sci. Technol. 2013, 7, 72. 23439162 10.1177/193229681300700109 PMC3692218

[81] S. Y. Ly , J. H. Lee , Ann. Biomed. Eng. 2009, 37, 2028. 19455422 10.1007/s10439-009-9714-1

[82] X. Li , M. Zhen , C. Zhou , R. Deng , T. Yu , Y. Wu , C. Shu , C. Wang , C. Bai , ACS Nano 2019, 13, 8597. 31314991 10.1021/acsnano.9b02050

[83] E. Demir , J. Bioenerg. Biomembr. 2021, 53, 25. 33411205 10.1007/s10863-020-09861-5

[84] E. Demir , A. Aslan , J. Food Biochem. 2020, 44, e13470. 32914898 10.1111/jfbc.13470

[85] R. Bal , G. Türk , M. Tuzcu , O. Yilmaz , I. Ozercan , T. Kuloglu , S. Gür , V. S. Nedzvetsky , A. A. Tykhomyrov , G. V. Andrievsky , G. Baydas , M. Naziroglu , Toxicology 2011, 282, 69. 21163323 10.1016/j.tox.2010.12.003

[86] M. Tanaka , Y. Sato , H. Haniu , H. Nomura , S. Kobayashi , S. Takanashi , M. Okamoto , T. Takizawa , K. Aoki , Y. Usui , A. Oishi , H. Kato , N. Saito , PLoS One 2017, 12, e0172601. 28235026 10.1371/journal.pone.0172601 PMC5325283

[87] M. Tanaka , Y. Sato , M. Zhang , H. Haniu , M. Okamoto , K. Aoki , T. Takizawa , K. Yoshida , A. Sobajima , T. Kamanaka , H. Kato , N. Saito , Nanomaterials 2017, 7, 46. 10.3390/nano7020046 PMC5333031 28336879

[88] K. Aoki , N. Ogihara , M. Tanaka , H. Haniu , N. Saito , J. Mater. Chem. B 2020, 8, 9227. 32935730 10.1039/d0tb01440k

[89] A. Sobajima , H. Haniu , H. Nomura , M. Tanaka , T. Takizawa , T. Kamanaka , K. Aoki , M. Okamoto , K. Yoshida , J. Sasaki , K. Ajima , C. Kuroda , H. Ishida , S. Okano , K. Ueda , H. Kato , N. Saito , Int. J. Nanomed. 2019, 14, 6465. 10.2147/IJN.S208129 PMC6698589 31616140

[90] X. Deng , G. Jia , H. Wang , H. Sun , X. Wang , S. Yang , T. Wang , Y. Liu , Carbon 2007, 45, 1419.

[91] S. Tang , Y. Tang , L. Zhong , K. Murat , G. Asan , J. Yu , R. Jian , C. Wang , P. Zhou , J. Appl. Toxicol. 2012, 32, 900. 22760929 10.1002/jat.2748

[92] J. E. Ferguson , S. M. Andrew , C. J. Jones , P. J. August , Br J Dermatol 1997, 137, 405. 9349338

[93] M. Vitiello , B. Echeverria , P. Romanelli , A. Abuchar , F. Kerdel , J. Clin. Aesthet. Dermatol. 2010, 3, 54. PMC2921756 20725558

[94] G. Forte , F. Petrucci , A. Cristaudo , B. Bocca , Sci. Total Environ. 2009, 407, 5997. 19766292 10.1016/j.scitotenv.2009.08.034

[95] N. Kluger , D. Douvin , F. Dupuis‐Fourdan , J. M. Doumecq‐Lacoste , V. Descamps , Ann Dermatol Venereol 2017, 144, 776. 29126557 10.1016/j.annder.2017.10.006

[96] F. J. Paprottka , S. Bontikous , J. A. Lohmeyer , D. Hebebrand , Plast. Reconstr. Surg. Glob. Open. 2014, 2, e114. 25289308 10.1097/GOX.0000000000000055 PMC4174140

[97] K. Hara , K. Aoki , Y. Usui , M. Shimizu , N. Narita , N. Ogihara , K. Nakamura , N. Ishigaki , K. Sano , H. Haniu , H. Kato , N. Nishimura , Y. A. Kim , S. Taruta , N. Saito , Mater. Today 2011, 14, 434.

[98] H. Nomura , S. Takanashi , M. Tanaka , H. Haniu , K. Aoki , M. Okamoto , S. Kobayashi , T. Takizawa , Y. Usui , A. Oishi , H. Kato , N. Saito , Sci. Rep. 2015, 5, 14314. 26388047 10.1038/srep14314 PMC4585697

[99] M. Sano , M. Izumiya , H. Haniu , K. Ueda , K. Konishi , H. Ishida , C. Kuroda , T. Uemura , K. Aoki , Y. Matsuda , N. Saito , Nanomaterials 2020, 10, 1374. 10.3390/nano10071374 PMC7407296 32674394

[100] A. Figarol , J. Pourchez , D. Boudard , V. Forest , C. Akono , J. M. Tulliani , J. P. Lecompte , M. Cottier , D. Bernache‐Assollant , P. Grosseau , Toxicol. In Vitro 2015, 30, 476. 26381085 10.1016/j.tiv.2015.09.014

[101] L. Ma‐Hock , V. Strauss , S. Treumann , K. Kuttler , W. Wohlleben , T. Hofmann , S. Groters , K. Wiench , B. van Ravenzwaay , R. Landsiedel , Part. Fibre Toxicol. 2013, 10, 23. 23773277 10.1186/1743-8977-10-23 PMC3720229

[102] A. Magrez , S. Kasas , V. Salicio , N. Pasquier , J. W. Seo , M. Celio , S. Catsicas , B. Schwaller , L. Forro , Nano Lett. 2006, 6, 1121. 16771565 10.1021/nl060162e

[103] J. Muller , F. Huaux , N. Moreau , P. Misson , J. F. Heilier , M. Delos , M. Arras , A. Fonseca , J. B. Nagy , D. Lison , Toxicol. Appl. Pharmacol. 2005, 207, 221. 16129115 10.1016/j.taap.2005.01.008

[104] E. Di Ianni , J. S. Erdem , P. Moller , N. M. Sahlgren , S. S. Poulsen , K. B. Knudsen , S. Zienolddiny , A. T. Saber , H. Wallin , U. Vogel , N. R. Jacobsen , Part. Fibre Toxicol. 2021, 18, 25. 34301283 10.1186/s12989-021-00413-2 PMC8299626

[105] U. C. Nygaard , J. S. Hansen , M. Samuelsen , T. Alberg , C. D. Marioara , M. Lovik , Toxicol. Sci. 2009, 109, 113. 19293371 10.1093/toxsci/kfp057

[106] A. Erdely , T. Hulderman , R. Salmen , A. Liston , P. C. Zeidler‐Erdely , D. Schwegler‐Berry , V. Castranova , S. Koyama , Y. A. Kim , M. Endo , P. P. Simeonova , Nano Lett. 2009, 9, 36. 19049393 10.1021/nl801828z

[107] H. Haniu , N. Saito , Y. Matsuda , Y. A. Kim , K. C. Park , T. Tsukahara , Y. Usui , K. Aoki , M. Shimizu , N. Ogihara , K. Hara , S. Takanashi , M. Okamoto , N. Ishigaki , K. Nakamura , H. Kato , Int. J. Nanomed. 2011, 6, 3295. 10.2147/IJN.S26573 PMC3252677 22228997

[108] H. J. Park , M. Park , J. Y. Chang , H. Lee , Nanotechnology 2008, 19, 335702. 21730628 10.1088/0957-4484/19/33/335702

[109] C. Kuroda , H. Haniu , K. Ajima , M. Tanaka , A. Sobajima , H. Ishida , T. Tsukahara , Y. Matsuda , K. Aoki , H. Kato , N. Saito , Nanomaterials 2016, 6, 219. 10.3390/nano6110219 PMC5245756 28335347

[110] C. Kuroda , K. Ueda , H. Haniu , H. Ishida , S. Okano , T. Takizawa , A. Sobajima , T. Kamanaka , K. Yoshida , M. Okamoto , T. Tsukahara , Y. Matsuda , K. Aoki , H. Kato , N. Saito , Int. J. Nanomed. 2018, 13, 6079. 10.2147/IJN.S172493 PMC6179726 30323595

[111] Y. Sakamoto , D. Nakae , N. Fukumori , K. Tayama , A. Maekawa , K. Imai , A. Hirose , T. Nishimura , N. Ohashi , A. Ogata , J. Toxicol. Sci. 2009, 34, 65. 19182436 10.2131/jts.34.65

[112] A. Takagi , A. Hirose , M. Futakuchi , H. Tsuda , J. Kanno , Cancer Sci. 2012, 103, 1440. 22537085 10.1111/j.1349-7006.2012.02318.x PMC3569866

[113] I. Marangon , C. Menard‐Moyon , J. Kolosnjaj‐Tabi , M. L. Beoutis , L. Lartigue , D. Alloyeau , E. Pach , B. Ballesteros , G. Autret , T. Ninjbadgar , D. F. Brougham , A. Bianco , F. Gazeau , Adv. Funct. Mater. 2014, 24, 7173.

[114] A. Rodriguez‐Galvan , M. Rivera , P. Garcia‐Lopez , L. A. Medina , V. A. Basiuk , J. Cell. Mol. Med. 2020, 24, 3779. 32154648 10.1111/jcmm.15065 PMC7171414

[115] B. Sitharaman , K. R. Kissell , K. B. Hartman , L. A. Tran , A. Baikalov , I. Rusakova , Y. Sun , H. A. Khant , S. J. Ludtke , W. Chiu , S. Laus , E. Toth , L. Helm , A. E. Merbach , L. J. Wilson , Chem. Commun. 2005, 31, 3915. 10.1039/b504435a 16075070

[116] S. T. Yang , W. Guo , Y. Lin , X. Y. Deng , H. F. Wang , H. F. Sun , Y. F. Liu , X. Wang , W. Wang , M. Chen , Y. P. Huang , Y. P. Sun , J. Phys. Chem. C 2007, 111, 17761.

[117] B. Czarny , D. Georgin , F. Berthon , G. Plastow , M. Pinault , G. Patriarche , A. Thuleau , M. M. L'Hermite , F. Taran , V. Dive , ACS Nano 2014, 8, 5715. 24853551 10.1021/nn500475u

[118] M. Jaymand , Y. D. Taghipour , A. Rezaei , H. Derakhshankhah , M. F. Abazari , H. Samadian , M. R. Hamblin , Coord. Chem. Rev. 2021, 440, 213974.

[119] Z. Liu , C. Davis , W. B. Cai , L. He , X. Y. Chen , H. J. Dai , Proc. Natl. Acad. Sci. U. S. A. 2008, 105, 1410. 18230737 10.1073/pnas.0707654105 PMC2234157

[120] J. W. Kang , F. T. Nguyen , N. Lue , R. R. Dasari , D. A. Heller , Nano Lett. 2012, 12, 6170. 23151070 10.1021/nl302991y PMC3561480

[121] L. N. Golubewa , T. A. Kulahava , Y. S. Leonik , M. V. Shuba , G. N. Semenkova , J. Appl. Spectrosc. 2021, 88, 77.

[122] E. N. Golubewa , M. V. Shuba , M. V. Vasilieu , T. A. Kulahava , J. Appl. Spectrosc. 2019, 85, 1121.

[123] L. Golubewa , T. Kulahava , Y. Kunitskaya , P. Bulai , M. Shuba , R. Karpicz , Biochem. Biophys. Res. Commun. 2020, 529, 647. 32736687 10.1016/j.bbrc.2020.06.100

[124] S. Kobayashi , S. Tsuruoka , Y. Usui , H. Haniu , K. Aoki , S. Takanashi , M. Okamoto , H. Nomura , M. Tanaka , S. Aiso , M. Saito , H. Kato , N. Saito , NPG Asia Mater. 2015, 7, e203.

[125] M. Takasaka , S. Kobayashi , Y. Usui , H. Haniu , S. Tsuruoka , K. Aoki , N. Saito , Toxics 2021, 9, 331. 34941765 10.3390/toxics9120331 PMC8705935

[126] C. Kuroda , K. Ajima , K. Ueda , A. Sobajima , K. Yoshida , T. Kamanaka , J. Sasaki , H. Ishida , H. Haniu , M. Okamoto , K. Aoki , H. Kato , N. Saito , Nano Today 2021, 36, 101018.

[127] K. Mitsumori , H. Koizumi , T. Nomura , S. Yamamoto , Toxicol. Pathol. 1998, 26, 520. 9715511 10.1177/019262339802600408

[128] S. Takanashi , K. Hara , K. Aoki , Y. Usui , M. Shimizu , H. Haniu , N. Ogihara , N. Ishigaki , K. Nakamura , M. Okamoto , S. Kobayashi , H. Kato , K. Sano , N. Nishimura , H. Tsutsumi , K. Machida , N. Saito , Sci. Rep. 2012, 2, 498. 22787556 10.1038/srep00498 PMC3391660

[129] N. Handa , K. Ishii , Y. Matsui , Y. Ando , Ebiomedicine 2015, 2, 1211. 26501120 10.1016/j.ebiom.2015.07.011 PMC4588369

[130] M. Sato , Y. Ochiai , S. Kijima , N. Nagai , Y. Ando , M. Shikano , Y. Nomura , CPT: Pharmacometrics Syst. Pharmacol. 2017, 6, 413. 28568566 10.1002/psp4.12203 PMC5529733

[131] S. Takahashi , K. Iwasaki , H. Shirato , M. M. Ho , M. Umezu , J. Artif. Organs 2021, 24, 90. 33079285 10.1007/s10047-020-01216-6 PMC7889561

[132] J. J. Callaghan , J. C. Albright , D. D. Goetz , J. P. Olejniczak , R. C. Johnston , J. Bone Jt. Surg., Am. Vol. 2000, 82A, 487. 10.2106/00004623-200004000-00004 10761939

[133] U. Kesteris , K. Hardinge , T. Ilchmann , H. Wingstrand , J. Arthroplasty 2003, 18, 10. 10.1054/arth.2003.50011 12555176

[134] S. Tarasevicius , O. Robertsson , U. Kesteris , R. J. Kalesinskas , H. Wingstrand , Acta Orthop. 2008, 79, 489. 18766481 10.1080/17453670710015472

[135] J. M. Wilkinson , A. J. Hamer , I. Stockley , R. Eastell , J. Orthop. Res. 2005, 23, 520. 15885470 10.1016/j.orthres.2004.11.005

[136] A. M. Kandahari , X. L. Yang , K. A. Laroche , A. S. Dighe , D. F. Pan , Q. J. Cui , Bone Res. 2016, 4, 16014. 27468360 10.1038/boneres.2016.14 PMC4941197

[137] J. Dahl , F. Snorrason , L. Nordsletten , S. M. Rohrl , Acta Orthop. 2013, 84, 360. 23795579 10.3109/17453674.2013.810516 PMC3768034

[138] J. Gallo , M. Slouf , S. B. Goodman , J. Biomed. Mater. Res., Part B 2010, 94B, 171. 10.1002/jbm.b.31638 20524192

[139] N. W. Emms , I. Stockley , A. J. Hamer , J. M. Wilkinson , J. Bone Jt. Surg., Br. Vol. 2010, 92B, 856. 10.1302/0301-620X.92B6.23666 20513885

[140] P. E. Purdue , P. Koulouvaris , H. G. Potter , B. J. Nestor , T. A. Sculco , Clin. Orthop. Relat. Res. 2007, 454, 251. 16980902 10.1097/01.blo.0000238813.95035.1b

[141] S. B. Goodman , J. Gallo , J. Clin. Med. 2019, 8, 2091.

[142] O. K. Muratoglu , C. R. Bragdon , D. O. O'Connor , M. Jasty , W. H. Harris , R. Gul , F. McGarry , Biomaterials 1999, 20, 1463. 10458559 10.1016/s0142-9612(99)00039-3

[143] O. K. Muratoglu , C. R. Bragdon , D. O'Connor , R. S. Perinchief , D. M. Estok , M. Jasty , W. H. Harris , J. Arthroplasty 2001, 16, 24. 11742447 10.1054/arth.2001.28376

[144] S. A. Hanna , L. Somerville , R. W. McCalden , D. D. Naudie , S. J. MacDonald , Bone Joint. J. 2016, 98B, 28. 10.1302/0301-620X.98B1.36527 26733512

[145] Y. Takaoka , K. Goto , J. Tamura , Y. Okuzu , T. Kawai , Y. Kuroda , K. Orita , S. Matsuda , Bone Joint J. 2021, 103B, 1604. 10.1302/0301-620X.103B10.BJJ-2020-2298.R2 34587810

[146] G. G. Roedel , B. J. Kildow , D. S. Sveom , K. L. Garvin , Bone Joint J. 2021, 103B, 78. 10.1302/0301-620X.103B7.BJJ-2020-2443.R1 34192900

[147] H. Prock‐Gibbs , C. A. Pumilia , T. Meckmongkol , J. Lovejoy , A. Mumith , M. Coathup , J. Bone Jt. Surg., Am. Vol. 2021, 103, 728. 10.2106/JBJS.20.01086 33411465

[148] J. van Loon , D. Hoornenborg , I. Sierevelt , K. T. M. Opdam , G. Kerkhoffs , D. Haverkamp , World J. Orthop. 2020, 11, 442. 33134107 10.5312/wjo.v11.i10.442 PMC7582113

[149] S. Braun , S. Jaeger , R. Sonntag , S. Schroeder , J. P. Kretzer , Materials 2020, 13, 1854. 10.3390/ma13081854 PMC7215625 32326506

[150] A. Klasan , T. Neri , A. Sen , B. F. El‐Zayat , T. Efe , M. Lahner , T. J. Heyse , Technol. Health Care 2020, 28, 415. 31796715 10.3233/THC-191896

[151] B. Feng , Y. Ren , S. L. Cao , J. Lin , J. Jin , W. W. Qian , X. S. Weng , J. Orthop. Surg. Res. 2019, 14, 388. 31775827 10.1186/s13018-019-1410-8 PMC6882238

[152] R. B. C. Treacy , J. P. Holland , J. Daniel , H. Ziaee , D. J. W. McMinn , Bone Joint Res. 2019, 8, 443. 31728182 10.1302/2046-3758.810.BJR-2019-0060.R1 PMC6825046

[153] J. Furmanski , M. Anderson , S. Bal , A. S. Greenwald , D. Halley , B. Penenberg , M. Ries , L. Pruitt , Biomaterials 2009, 30, 5572. 19643471 10.1016/j.biomaterials.2009.07.013

[154] M. P. Ast , T. K. John , A. Labbisiere , N. Robador , A. G. Della Valle , J. Arthroplasty 2014, 29, 1231. 24444569 10.1016/j.arth.2013.12.022

[155] T. J. Blumenfeld , H. A. McKellop , T. P. Schmalzried , F. Billi , J. Arthroplasty 2011, 26, 666.e5. 10.1016/j.arth.2010.07.009 20851563

[156] W. Waewsawangwong , S. B. Goodman , J. Arthroplasty 2012, 27, 323.e1. 10.1016/j.arth.2011.04.010 21601415

[157] D. Hara , Y. Nakashima , T. Yamamoto , S. Higashihara , M. Todo , M. Hirata , M. Akiyama , Y. Iwamoto , J. Mech. Behav. Biomed. Mater. 2013, 28, 206. 23995342 10.1016/j.jmbbm.2013.08.003

[158] S. S. Tower , J. H. Currier , B. H. Currier , K. A. Lyford , D. W. Van Citters , M. B. Mayor , J. Bone Jt. Surg., Am. Vol. 2007, 89A, 2212. 10.2106/JBJS.F.00758 17908898

[159] J. J. Jauregui , Q. Naziri , T. P. Pierce , R. K. Elmallah , J. J. Cherian , R. E. Delanois , M. A. Mont , Int. Orthop. 2016, 40, 681. 26130285 10.1007/s00264-015-2841-4

