# Supplementary material for: Future Prospects for Clinical Applications of Nanocarbons Focusing on Carbon Nanotubes
Source: Adv Sci (Weinh). 2022 Jun 26;9(24):2201214. doi: 10.1002/advs.202201214 (PMC9404397; doi:10.1002/advs.202201214)
Supplement: Supplementary file 1 — Supporting Information [file ADVS-9-2201214-s002.pdf]

ADVANCED SCIENCE
Open Access

Supporting Information

for *Adv. Sci.*, DOI 10.1002/advs.202201214

Future Prospects for Clinical Applications of Nanocarbons Focusing on Carbon Nanotubes

Naoto Saito, Hisao Haniu, Kaoru Aoki, Naoyuki Nishimura and Takeshi Uemura*

Road to the Clinical Use of Nanocarbon Biomaterials

Naoto Saito, Hisao Haniu, Kaoru Aoki, Naoyuki Nishimura, Takeshi Uemura*

N. Saito, H. Haniu, N. Nishimura, T. Uemura

Institute for Biomedical Sciences, Interdisciplinary Cluster for Cutting Edge Research, Shinshu University, 3-1-1 Asahi, Matsumoto, Nagano 390-8621, Japan

K. Aoki

Department of Applied Physical Therapy, Shinshu University School of Health Sciences, 3-1-1 Asahi, Matsumoto, Nagano 390-8621, Japan

T. Uemura

Division of Gene Research, Research Center for Supports to Advanced Science, Shinshu University, 3-1-1 Asahi, Matsumoto, Nagano 390-8621, Japan

* Corresponding author

E-mail address: saitoko@shinshu-u.ac.jp

Supporting Information

Supplementary Video 1

A rat iliac lymph node afferent lymphatic vessel was isolated from the animal. Its ends were inserted into a glass micropipette in the organ chamber and ligated to prepare an isolated lymphatic vessel perfusion specimen. After inducing the lymphatic vessel to contract spontaneously and confirming the presence of intact lymphatic vessel endothelial cells and smooth muscle cells, nanomaterial dispersion was perfused into the isolated lymphatic vessel by the force of the vessel's spontaneous contractions. (a) Video of spontaneous contractions of the lymphatic vessel prior to MWCNT perfusion. Spontaneous contractions of the lymphatic vessel with Krebs' solution in the cavity prior to MWCNT perfusion. The lymphatic vessel's diameter changed and the valve moved in synchronization with the spontaneous contractions. (b) Video of spontaneous contractions of the lymphatic vessel being perfused with MWCNTs. Spontaneous contractions of the lymphatic vessel being perfused with MWCNTs (1 mg/mL). The black MWCNTs were found to flow in synchronization with the motions of spontaneous contractions. When the lymphatic vessel contracted, the MWCNTs moved faster anteriorly and posteriorly to the valve in the vessel. When the lymphatic vessel dilated, the particles flew across the valve in the lymphatic vessel. During the contraction and dilation of the lymphatic vessel, the valve moved across the center of the lymphatic vessel. The intimal inflow (IN) is shown on the right side of the screen, and the outflow (OUT) is on the left side. Images were taken at 400x magnification and an imaging rate of 500 frames per second (fps) using a high-speed camera (FAST CAM AX50, Photron, Tokyo, Japan), which were reproduced at 250 fps (approximately 0.5 times normal speed). Rerun with permission from Ref. [122]
